# 

**Published:** 1888-03

**Authors:** 


### TABLE OF CONTENTS.

### ORIGINAL AND SELECTED ARTICLES.

Duality of the brain—a theory of mind-reading and slate-writing, by R. C. Word, M. D... 81
Ipecacuanha, by L. B. Bouchelle, M.D., Thomasville, Ga. ... 89
Obstetric anæsthesia, by Dr. N. M. Dodson, Berlin, Wis ... 92
Peculiarities in the therapeutic action of nux vomica in different individuals, by Chas. Chassaignac, M. D., New Orleans ... 94
The use of salt in the hygiene and therapeutics of the skin, by H. G. Piffard, M. D., of New York ... 96

### ABSTRACTS AND GLEANINGS.

Carbuncle ... 97
Hydrastis canadensis in uterine hemorrhage.. 99
Antipyrin as a hæmostatic; how to administer chloroform; Bacteriology and practical medicine ... 100
Intubation or tracheotomy; antipyrin as a uterine sedative ... 102
Relative value of antipyrin and antifebrin; Some remedies in diphtheria ... 103
Sulphate of sparteine; diphtheria of the ear.. 104
Medical aphorisms; salicylate of sodium in whooping-cough ... 105
Laparotomy in peritonitis; treatment of warts by internal administration of arsenic; artificial ripening of cataracts ... 106
Ephedrine, a new mydriatic; the finishing touch; poisoned by flannel underwear ... 107
Salol in typhoid fever; another cure for consumption; law of proportion of sexes; theine in rheumatism [illegible] ... 108
Prophylaxis of [illegible] uterine hemorrhage; Detention of me[illegible] bodies with the magnet; chloral hyd[illegible] in rabies; removing particles from the eye; canadol, a new local anæsthetic ... 109
Some suggestions on the pathogenesis of yellow fever; glycerine in enemata; salicylate of sodium in facial neuralgia; a remedy for neuralgia; arnica; cocaine hydrychlorate... 110

### SCIENTIFIC ITEMS.

Great war ships and forts; the nose the source of all our woes ... 111
Solidification of liquids by pressure; the chrotograph ... 112

### PRACTICAL NOTES AND FORMULÆ.

For chronic gonorrhœa; delirium of typhoid fever; pyæmia; acute tonsilitis; acute rheumatism ... 113

### EDITORIALS AND MISCELLANEOUS.

Editorial brevities ... 114
Hydrophobia; beautiful token; the William E. Jenks memorial prize ... 115
"Tuto, cito et jucunde." ... 116
Quack advertisements in religious newspapers ... 116
The commencement exercises ... 117
Sanders & Son's eucalyptol; books and pamphlets received ... 119
Special notes ... 120

